# Adherence to a healthy Nordic diet and risk of type 2 diabetes among men: the Kuopio Ischaemic Heart Disease Risk Factor Study

**DOI:** 10.1007/s00394-021-02569-1

**Published:** 2021-04-27

**Authors:** Hanna-Mari Tertsunen, Sari Hantunen, Tomi-Pekka Tuomainen, Jyrki K. Virtanen

**Affiliations:** Institute of Public Health and Clinical Nutrition, University of Eastern Finland, P.O. Box 1627, 70211 Kuopio, Finland

**Keywords:** Baltic Sea Diet Score, Nordic diet, Type 2 diabetes, Population study, Prospective study

## Abstract

**Purpose:**

To investigate the association between healthy Nordic diet and risk of type 2 diabetes (T2D) in middle-aged and older men from eastern Finland.

**Methods:**

A total of 2332 men aged 42–60 years and free of T2D at baseline in 1984–1989 were included. Diet was assessed with 4-day food records at baseline and the healthy Nordic diet score was calculated based on a modified Baltic Sea Diet Score. T2D diagnosis was based on self-administered questionnaires, fasting and 2-h oral glucose tolerance test blood glucose measurements, or by record linkage to national health registries. Cox proportional hazards regression and analysis of covariance were used for analyses.

**Results:**

During the mean follow-up of 19.3 years, 432 men (18.5%) were diagnosed with T2D. The multivariable-adjusted hazard ratio for T2D in the lowest vs. the highest quartile of the healthy Nordic diet score was 1.35 (95% CI 1.03–1.76) (*P* trend across quartiles 0.028). Lower adherence to healthy Nordic diet was also associated with higher plasma glucose and insulin concentrations.

**Conclusions:**

In this prospective population-based cohort study among middle-aged and older men from eastern Finland, lower adherence to healthy Nordic diet was associated with higher risk of T2D and higher plasma glucose and serum insulin concentrations.

**Supplementary Information:**

The online version contains supplementary material available at 10.1007/s00394-021-02569-1.

## Introduction

Lifestyle factors and diet have been associated with the risk of type 2 diabetes (T2D) [1, 2]. Even though some individual dietary factors, such as whole grains and fiber [3], have been associated with lower risk of T2D, diet consists of combinations of numerous nutrients and foods. Dietary scores are used to measure the healthiness of the whole diet by considering the cumulative effects and interactions between several food items and nutrients [4]. The Mediterranean diet is one example of the diets that have been associated with lower T2D risk [5] but due to cultural and geographical factors adherence to it is relatively poor in Nordic countries [6]. For example, in Finland, Baltic Sea Diet Score was developed to characterize a healthy Nordic diet based on typical Finnish foods and is characterized by high consumption of berries and fruits, whole grains, vegetables, rapeseed oil, fish, low-fat dairy, and low consumption of processed meat and alcohol [7].

A healthy Nordic diet has been reported to beneficially associate with T2D risk factors such as abdominal obesity [8] and obesity-related markers of inflammation [9] in observational studies, and in randomized controlled trials with lipid profile in hypercholesterolemic subjects [10] and with inflammation markers [11]. However, the association between adherence to a healthy Nordic diet and incidence of T2D is less investigated and the findings are inconsistent. A prospective study including two independent Finnish studies did not find an association between a healthy Nordic diet and incidence of T2D [12] but a larger cohort study from Denmark found an inverse association between adherence to a healthy Nordic diet and risk of T2D [13].

Because of the limited research data about adherence to a healthy Nordic diet and risk of glucose metabolism disturbances, our aim was to examine the association between a healthy Nordic diet, based on a modified Baltic Sea Diet Score, and risk of incident T2D in the Kuopio Ischaemic Heart Disease Risk Factor Study (KIHD), a population of middle-aged and older men from eastern Finland.

## Materials and methods

### Study population

The KIHD was designed to investigate risk factors for cardiovascular diseases, atherosclerosis, and related outcomes in a prospective, population-based sample of men from eastern Finland [14]. The baseline examinations were carried out in 1984–1989. A total of 2682 men (83% of those eligible) who were 42, 48, 54 or 60 years old at baseline participated in the examinations. The baseline characteristics of the entire study population have been described elsewhere [15]. The KIHD protocol was approved by the Research Ethics Committee of the University of Kuopio. All subjects gave written informed consent for participation. Subjects with history of T2D (*n* = 167) at baseline, impaired fasting glucose (defined as fasting plasma glucose 6.1–6.9 mmol/L, *n* = 127) or unknown diabetes status (*n* = 38) at baseline, or with missing data on dietary intakes (*n* = 18) were excluded, leaving 2332 men for the analyses. Information on plasma glucose and serum insulin was available for 2285 subjects.

### Assessment of dietary intakes

Consumption of foods at baseline was assessed with an instructed food recording of four days, of which one was a weekend day, by household measures. A picture book of common foods and dishes was used to help in estimation of portion sizes. The picture book contained 126 most common foods and drinks consumed in Finland during the 1980s, and for each food item, the participant could choose from 3 to 5 commonly used portion sizes or describe the portion size in relation to those in the book. To further improve accuracy, instructions were given and completed food records were checked by a nutritionist together with the participant. Nutrient intakes were estimated using the NUTRICA^®^ 2.5 software (Social Insurance Institution, Turku, Finland). The databank of the software is mainly based on Finnish values of nutrient composition of foods.

### Healthy Nordic diet score

The original Baltic Sea Diet Score consists of nine components, of which six are food groups and three represent nutrients [7]. The food items have been selected based on the traditional food culture in Finland. However, due to the lack of information on certain food items in our database, the contents in the food groups in our study are not identical to the original Baltic Sea Diet Score. The original Baltic Sea Diet Score components and those used in the current study are presented in Supplemental Table 1. The score was calculated according to quartiles of consumption for each score component. For the positive score components, the lowest quartile was given 0 points, the second one 1 point, the third one 2 points and the highest quartile of intake 3 points. For the negative score components, points were given in reverse order, except for alcohol, which was given 0–1 point (one point was given if the ethanol intake was < 20 g/day, otherwise zero points were given). The points given for each component were summed up to obtain the overall score. The resulting score ranged from 0 to 25. The higher the score, the higher the adherence to a healthy Nordic diet. In the analysis, the score was categorized into quartiles. The lowest quartile included scores ≤ 9, the two middle quartiles included scores 10–12 and 13–14 and the highest quartile included scores ≥ 15.

### Health examination and measurements

Venous blood samples were collected between 8 and 10 AM at the baseline examinations. Subjects were instructed to abstain from ingesting alcohol for three days and from smoking and eating for 12 h prior to giving the sample. Detailed descriptions of the determination of serum lipids and lipoproteins [16], blood pressure [16], assessment of medical history and medications at baseline [16], family history of diseases [16], smoking [16], alcohol intake [16], and physical activity [17], have been published. Hypertension was defined as blood pressure over 140/90 mmHg or medical treatment for hypertension. Serum C-reactive protein (CRP) was measured with an immunometric assay (Immulite High Sensitivity CRP Assay, DPC, Los Angeles, CA, USA) using clinical chemistry analyser Kone Specific (KONE Instruments Oy, Espoo, Finland). Plasma glucose was measured using a glucose dehydrogenase method after precipitation of proteins by trichloroacetic acid. Serum insulin was determined with a Novo Biolabs radioimmunoassay kit (Novo Nordisk, Bagsvaerd, Denmark). Body mass index (BMI) was computed as the ratio of weight in kilograms to the square of height in meters. Information on the medication use during the follow-up was obtained from the national Drug Prescription Registry at the Social Insurance Institute. Education and income were assessed by self-administered questionnaires. The family history of diabetes was defined as “yes”, if a first degree relative had diabetes history.

### Ascertainment of follow-up events

T2D was defined as a self-reported physician-set diagnosis of T2D and/or fasting plasma glucose ≥ 7.0 mmol/L or 2-h oral glucose tolerance test plasma glucose ≥ 11.1 mmol/L at re-examination rounds 4, 11 and 20 years after the baseline, or by record linkage to the national hospital discharge registry and to the Social Insurance Institution of Finland register for reimbursement of medicine expenses used for T2D for the entire study period until the end of follow-up in Dec 31, 2010. The 2-h oral glucose tolerance test was not done at the study baseline.

### Statistical analysis

Plasma glucose and serum insulin concentrations in quartiles of the healthy Nordic diet score were analyzed with analysis of covariance and linear regression. Cox proportional hazards regression models were used to estimate hazard ratios (HR) of T2D risk in quartiles of the score. The validity of the proportional hazards assumption was confirmed by using Schoenfeld residuals. Due to the low range of values in the score, the number of participants in each quartile is not even. The analyses were adjusted for relevant covariates: The Model 1 included age (years), examination year and energy intake (kcal/day). The multivariable Model 2 included Model 1 and smoking (never smoker, previous smoker, current smoker < 20 cigarettes/day and current smoker ≥ 20 cigarettes/day), BMI (kg/m^2^), leisure-time physical activity (kcal/day), education (years), marital status (married vs other), income (euros/year) and family history of diabetes (yes/no). All quantitative variables were entered in the models as continuous variables. Cohort mean was used to replace missing values in covariates (< 2.4%). Tests of linear trend were conducted by assigning the median values for each category of exposure variable and treating those as a single continuous variable*.* All *P* values were 2-tailed (*α* = 0.05). Data were analysed using SPSS 25.0 for Windows (Armonk, NY: IBM Corp. US).

## Results

### Baseline characteristics of the study population

The baseline characteristics for the 2332 participants are presented in Table 1. Participants who had lower adherence to healthy Nordic diet had lower leisure-time physical activity and education, and they were less likely to use hypertension and cholesterol medications at baseline. They also had higher alcohol intake, diastolic blood pressure, total, LDL and HDL cholesterol concentrations and CRP concentration and they were more likely smokers and less likely married and less likely to have history of diabetes in family compared to the participants with higher adherence to healthy Nordic diet.

**Table 1:** Baseline characteristics of the 2332 participants according to quartiles of the healthy Nordic diet score

	Quartile of the healthy Nordic diet score (score)			
Characteristic	1 (≤ 9)	2 (10–12)	3 (13–14)	4 (≥ 15)
Number of subjects	664	438	607	623
Age (years)	52.8 ± 5.0^a^	52.8 ± 5.3	52.9 ± 5.0	53.2 ± 5.2
Body mass index (kg/m^2^)	26.6 ± 3.3	26.5 ± 3.4	26.7 ± 3.2	26.6 ± 3.3
Leisure-time physical activity (kcal/day)	108 ± 163	128 ± 150	150 ± 168	170 ± 196
Income (euros)	11,441 ± 8056	13,169 ± 8129	13,689 ± 8587	14,586 ± 8506
Education (years)	7.8 ± 2.8	8.7 ± 3.4	8.8 ± 3.7	9.4 ± 3.7
Energy intake (kcal/day)	2492 ± 619	2447 ± 624	2458 ± 624	2428 ± 568
Alcohol intake (g/week)	95 ± 158	71 ± 101	76 ± 162	49 ± 82
Mean systolic blood pressure	134 ± 16.4	133 ± 15.6	133 ± 17.3	132 ± 16.1
Mean diastolic blood pressure	89 ± 10.7	88 ± 9.7	89 ± 10.5	88 ± 10.3
C-reactive protein (mmol/L)	2.77 ± 5.2	2.42 ± 3.8	2.20 ± 3.3	1.94 ± 3.5
Total cholesterol (mmol/L)	6.03 ± 1.1	6.02 ± 1.1	5.82 ± 1.0	5.77 ± 1.0
LDL cholesterol (mmol/L)	4.16 ± 1.1	4.11 ± 1.1	3.98 ± 1.0	3.96 ± 1.0
HDL cholesterol (mmol/L)	1.32 ± 0.3	1.31 ± 0.3	1.30 ± 0.3	1.27 ± 0.3
Triglycerides (mmol/L)	1.24 ± 0.8	1.30 ± 0.8	1.24 ± 0.7	1.30 ± 0.7
Use of hypertension medication at baseline (%)	17.2	19.2	18.3	25.7
Use of hypertension medication during follow-up (%)	74.5	75.8	76.9	77.2
Use of cholesterol medication at baseline (%)	0	0.5	0.2	1.3
Use of cholesterol medication during follow-up (%)	49.4	50.2	51.4	51.8
Current smoker (%)	47.7	33.3	27.2	19.1
Diabetes history in family (%)	24.5	25.8	26.2	30.0
Cardiovascular disease history in family (%)	81.2	79.7	82.7	83.6
Marital status married (%)	80.4	88.8	88.5	91.3

^a^All values are means (SD) or percentages

### Risk of incident type 2 diabetes

**Fig. 1:**
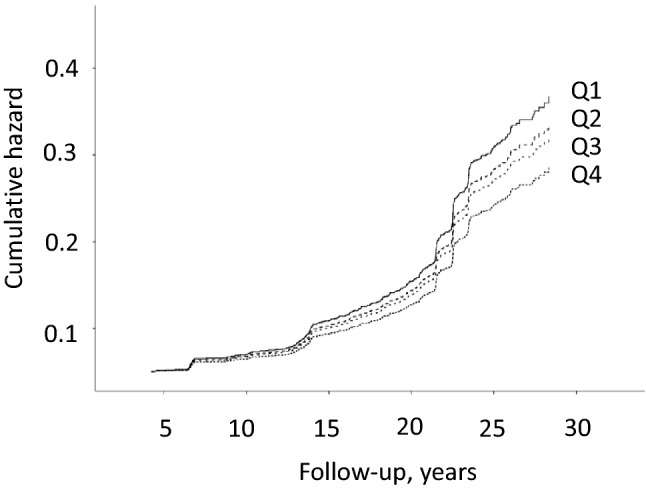
Cumulative hazard of type 2 diabetes (*n* = 432) according to the quartiles of the healthy Nordic diet score among 2332 men from the Kuopio Ischaemic Heart Disease Risk Factors Study. The model is adjusted for age (years), examination year, energy intake (kcal/day), smoking (never smoker, previous smoker, current smoker < 20 cigarettes/day and current smoker ≥ 20 cigarettes/day), body mass index (kg/m^2^), leisure-time physical activity (kcal/day), education (years), marital status (married vs other), income (euros/year), and family history of diabetes (yes/no)

During the average follow-up of 19.3 years (SD 6.6 years), 432 men (18.5%) experienced a T2D event. In the Model 1, those in the lowest vs. the highest quartile of the healthy Nordic diet score had 48% (95% CI 14–92%) higher risk of T2D event (*P* trend across quartiles 0.003) (Table 2). After further adjustment for potential confounders, those in the lowest vs. the highest quartile had 35% (95% CI 3–76%) higher risk of T2D event (*P* trend across quartiles 0.028) (Model 2, Table 2, Fig. 1). Each one unit decrease in the score was associated with 3.0% (95% CI 0.5–5.5%) higher risk of T2D (Table 2).

**Table 2:** Risk of type 2 diabetes in quartiles of the healthy Nordic diet score

	Quartile of the healthy Nordic diet score (score)				*P* for trend	Hazard ratio (95% confidence interval) for one unit decrease in the score
	1 (≤ 9) (*n* = 664)	2 (10–12) (*n* = 438)	3 (13–14) (*n* = 607)	4 (≥ 15) (*n* = 623)		
Number of events (% of subjects)	136 (20.5)	80 (18.3)	114 (18.8)	102 (17.3)		
Incidence rate/1000 person-years	11.11	9.54	9.44	8.35		
Model 1	1.48 (1.14–1.92)	1.22 (0.91–1.63)	1.17 (0.90–1.54)	1	0.003	1.039 (1.015–1.062)
Model 2	1.35 (1.03–1.76)	1.21 (0.90–1.63)	1.15 (0.88–1.51)	1	0.028	1.030 (1.005–1.055)

Model 1 is adjusted for age (years), examination year and energy intake (kcal/day)

Model 2 is adjusted for Model 1 plus smoking (never smoker, previous smoker, current smoker < 20 cigarettes/day and current smoker ≥ 20 cigarettes/day), body mass index (kg/m^2^), leisure-time physical activity (kcal/day), education (years), marital status (married vs other), income (euros/year), and family history of diabetes (yes/no)

### Plasma glucose and serum insulin

When we examined the associations with the major T2D risk factors, in the Model 1 adjusted for age, examination year and energy intake, lower adherence to healthy Nordic diet was associated with higher plasma glucose concentrations (Table 3). After further adjustment for potential confounders (Model 2), the association remained statistically significant (extreme-quartile difference 0.071 mg/L, 95% CI 0.026–0.116 mg/L). Similar, although weaker inverse association was observed with serum insulin concentrations (extreme-quartile difference 0.613 mU/L, 95% CI − 0.003–1.229 mU/L, *P* trend = 0.049, Model 2, Table 3).

**Table 3:** Concentrations of plasma glucose and serum insulin in quartiles of the healthy Nordic diet score

	Quartile of the healthy Nordic diet score (score)				*P* for trend	Change for one unit decrease in the score
	1 (≤ 9) (*n* = 647)	2 (10–12) (*n* = 432)	3 (13–14) (*n* = 596)	4 (≥ 15) (*n* = 610)		
Plasma glucose (mmol/L)						
Model 1	4.57 (4.54–4.60)	4.51 (4.47–4.54)	4.51 (4.48–4.55)	4.49 (4.46–4.52)	0.001	0.007 (0.003–0.011)
Model 2	4.56 (4.53–4.59)	4.51 (4.47–4.55)	4.51 (4.48–4.55)	4.49 (4.46–4.52)	0.003	0.007 (0.003–0.011)
Serum insulin (mU/L)						
Model 1	11.17 (10.70–11.64)	10.82 (10.24–11.39)	10.84 (10.35–11.33)	10.52 (10.04–11.01)	0.072	0.051 (0.013–0.115)
Model 2	11.14 (10.73–11.56)	10.91 (10.41–11.41)	10.79 (10.37–11.22)	10.53 (10.10–10.96)	0.049	0.053 (0.005–0.112)

Model 1 is adjusted for age (years), examination year and energy intake (kcal/day)

Model 2 is adjusted for Model 1 plus smoking (never smoker, previous smoker, current smoker < 20 cigarettes/day and current smoker ≥ 20 cigarettes/day), body mass index (kg/m^2^), leisure-time physical activity (kcal/day), education (years), marital status (married vs other), income (euros/year), and family history of diabetes (yes/no)

## Discussion

In this prospective population-based cohort study among middle-aged and older men from eastern Finland, lower adherence to healthy Nordic diet was associated with higher plasma glucose and serum insulin concentrations and with higher risk of T2D.

To our knowledge, there are only two studies that have earlier reported the association between a healthy Nordic diet and risk of T2D. A large cohort study of 55,060 participants aged between 50 and 64 from Denmark found an inverse association between adherence to a healthy Nordic diet measured with a healthy Nordic food index developed in Denmark and lower risk of T2D in both women and men [13]. In contrast, a prospective study including 6745 participants from two independent Finnish studies with mean ages ranging from 47 to 62 years did not find an association between a healthy Nordic diet, assessed with a Baltic Sea Diet Score, and incidence of T2D [12]. Both studies used self-administered semiquantitative food-frequency questionnaire to collect information about diet, instead of the 4-day food recording used in our study. The differences of the original Baltic Sea Diet Score, our modified score and the healthy Nordic food index are presented in Table 4. The healthy Nordic food index was based on intakes of fish, cabbage, rye bread, oatmeal, apples and pears, and root vegetables, but did not include some factors used in our score or in the original Baltic Sea Diet Score, such as dietary fat quality or dairy and meat intakes. It is interesting to note that the two rather differently composed scores to describe a healthy Nordic diet, our score and the Danish score, gave congruent associations with the risk of T2D, whereas the study using the original Baltic Sea Diet Score did not find an association. A partial explanation for the lack of association in the other Finnish study compared to our study may be the differences in diagnosis of T2D. They based the T2D diagnosis only on the national administrative registers, whereas we additionally considered the fasting and 2-h oral glucose tolerance test plasma glucose and serum insulin concentrations during the re-examination rounds 4, 11 and 20 years after the baseline, which also captures those cases that were not yet diagnosed by a physician. However, it is possible that none of the dietary scores that have been developed to describe a healthy Nordic diet can completely capture all potentially healthy foods used in the Nordic countries. For example, intake data on the commonly used rapeseed oil, a rich source of both omega-6 and omega-3 polyunsaturated fatty acids, is rarely available. Therefore, the choice of the potential components to include in the score is based on the availability of data and, in addition to the subjective decisions on what components to include, has an impact on how accurately a score describes a healthy Nordic diet and how well it can predict risk of disease.

**Table 4:** Components in different healthy Nordic diet scores

Original Baltic Sea Diet Score [7]	Score used in the current study	Healthy Nordic food index [34]
Berries, apples, pears	All fruits, berries	Fish
Tomato, cucumber, cabbage, roots, peas, lettuce	Roots, pulses, vegetables	Cabbages
Rye, oats, barley	Whole grains^a^	Whole-grain rye (rye bread)
Fat-free milk and milk < 2% fat	Fat-free milk and milk < 2% fat	Whole-grain oats (oatmeal)
Salmon, freshwater fish	Salmon, freshwater fish	Apples and pears
Beef, pork, processed meat products, sausages	Processed and unprocessed meat	Root vegetables
Total fat as a percentage of total energy intake	Total fat as a percentage of total energy intake	
Ratio of PUFA^b^ to SFA^c^ + trans-fatty acids	Ratio of PUFA to SFA + trans-fatty acids	
Ethanol	Ethanol	

^a^Excluding rice and pasta

^b^*PUFA* polyunsaturated fatty acids

^c^*SFA* saturated fatty acids

The associations of individual dietary factors with risk of T2D have been extensively studied [18, 19]. Higher intake of whole grains and fiber [20], low-fat dairy products, especially low-fat yogurt [21], and fruits and vegetables [22] have been associated with lower incidence of T2D, whereas higher intake of red meat, especially processed meat [23], and sugar sweetened beverages [24] have been associated with higher risk of developing T2D. Dietary fatty acid composition and risk of T2D have also earlier been widely studied in population studies. Total fat intake does not seem to associate with the risk of T2D, and there is inconclusive data about saturated fatty acid intake and risk of T2D [25]. Omega-6 polyunsaturated fatty acids, mostly linoleic acid, have been inversely associated with risk of T2D [26], whereas studies of omega-3 polyunsaturated fatty acid have been more controversial [27]. In the KIHD cohort, both omega-3 [28] and omega-6 [29] polyunsaturated fatty acids have an inverse association with risk of T2D. Studies have also reported an inverse association between light or moderate alcohol consumption and increased insulin sensitivity [30].

In our current study, healthy Nordic diet was associated with lower plasma glucose and serum insulin concentrations. Earlier studies have reported incoherent results of the association between a healthy Nordic diet and glucose metabolism indices. A meta-analysis of randomized controlled trials reported that a healthy Nordic diet improved serum insulin concentration [31]. Healthy Nordic diet has been also shown to improve fasting glucose level and insulin response, even when the changes in body weight are considered [32]. On the contrary, a randomized controlled SYSDIET intervention reported that a healthy Nordic diet had no effect on insulin sensitivity or glucose tolerance probably due to stable weight of the study participants [11].

The strengths of our study include the population-based cohort setting, comprehensive information about incident T2D events and viable confounding factors and no loss to follow-up. Limitations of our study are the possible random errors in food recording that would attenuate the true associations. Dietary intake was only assessed at baseline, which does not consider possible dietary changes during the long follow-up. Four-day food recording was used to assess dietary intakes, and even though it is considered to be the gold standard for dietary assessment, it has limitations with food items that are only occasionally consumed. For example, the representative intakes of fish and processed meat might have not been captured during the 4-day food recording. As an example, in the KIHD cohort the serum long-chain omega-3 PUFA concentration was inversely associated with risk of T2D, whereas intake of these fatty acids or fish was not [28]. Although the concentrations of the long-chain omega-3 PUFA are also affected by genetics and other non-dietary factors, intake of fish and the long-chain omega-3 PUFAs is the main determinant of the concentrations [33]. Therefore, such an objective biomarker of intake is less subject to random errors that are inherited in the subjective dietary assessment methods. Finally, due to the lack of information on some score components, we had to use a modified Baltic Sea Diet Score, so our findings are not directly comparable with the studies using the original score.

In conclusion, among middle-aged and older men from eastern Finland, lower adherence to a healthy Nordic diet was associated with higher risk of T2D and with higher plasma glucose and serum insulin concentrations. Due to the limited and conflicting data on the impact of a healthy Nordic diet on the risk of T2D, more research in diverse study populations is needed before strong conclusions can be drawn.

## Supplementary Information

Below is the link to the electronic supplementary material.

Supplementary file1 (DOCX 29 KB)

## Abbreviations

BMI: Body mass index
CI: Confidence interval
CRP: C-reactive protein
HDL: High-density lipoprotein
HR: Hazard ratio
KIHD: Kuopio Ischaemic Heart Disease Risk Factor Study
LDL: Low-density lipoprotein
PUFA: Polyunsaturated fatty acids
SFA: Saturated fatty acids
T2D: Type 2 diabetes

## References

[1] Tuomilehto J, Lindström J, Eriksson JG, Valle TT, Hämäläinen H, Ilanne-Parikka P, Keinänen-Kiukaanniemi S, Laakso M, Louheranta A, Rastas M, Salminen V, Uusitupa M (2001). Prevention of type 2 diabetes mellitus by changes in lifestyle among subjects with impaired glucose tolerance. N Engl J Med.

[2] Hu FB (2011). Globalization of diabetes: the role of diet, lifestyle, and genes. Diabetes Care.

[3] Montonen J, Knekt P, Järvinen R, Aromaa A, Reunanen A (2003). Whole-grain and fiber intake and the incidence of type 2 diabetes. Am J Clin Nutr.

[4] Waijers PMCM, Feskens EJM, Ocké MC (2007). A critical review of predefined diet quality scores. Br J Nutr.

[5] Romaguera D, Guevara M, Norat T, Langenberg C, Forouhi NG, Sharp S, Slimani N, Schulze MB, Buijsse B, Buckland G, Molina-Montes E, Sánchez MJ, Moreno-Iribas MC, Bendinelli B, Grioni S, van der Schouw YT, Arriola L, Beulens JW, Boeing H, Clavel-Chapelon F, Cottet V, Crowe FL, de Lauzon-Guillan B, Franks PW, Gonzalez C, Hallmans G, Kaaks R, Key TJ, Khaw K, Nilsson P, Overvad K, Palla L, Palli D, Panico S, Quirós JR, Rolandsson O, Romieu I, Sacerdote C, Spijkerman AMW, Teucher B, Tjonneland A, Tormo MJ, Tumino R, van der, A. D. L., Feskens EJM, Riboli E, Wareham NJ, (2011). Mediterranean diet and type 2 diabetes risk in the European Prospective Investigation into Cancer and Nutrition (EPIC) Study: the InterAct project. Diabetes Care.

[6] Sofi F, Abbate R, Gensini GF, Casini A (2010). Accruing evidence on benefits of adherence to the Mediterranean diet on health: an updated systematic review and meta-analysis. Am J Clin Nutr.

[7] Kanerva N, Kaartinen NE, Schwab U, Lahti-Koski M, Männistö S (2014). The Baltic Sea Diet Score: a tool for assessing healthy eating in Nordic countries. Public Health Nutr.

[8] Kanerva N, Kaartinen NE, Schwab U, Lahti-Koski M, Männistö S (2013). Adherence to the Baltic Sea diet consumed in the Nordic countries is associated with lower abdominal obesity. Br J Nutr.

[9] Kanerva N, Loo B, Eriksson JG, Leiviskä J, Kaartinen NE, Jula A, Männistö S (2014). Associations of the Baltic Sea diet with obesity-related markers of inflammation. Ann Med.

[10] Adamsson V, Reumark A, Fredriksson I, Hammarström E, Vessby B, Johansson G, Risérus U (2011). Effects of a healthy Nordic diet on cardiovascular risk factors in hypercholesterolaemic subjects: a randomized controlled trial (NORDIET). J Intern Med.

[11] Uusitupa M, Hermansen K, Savolainen MJ, Schwab U, Kolehmainen M, Brader L, Mortensen LS, Cloetens L, Johansson-Persson A, Onning G, Landin-Olsson M, Herzig K, Hukkanen J, Rosqvist F, Iggman D, Paananen J, Pulkki KJ, Siloaho M, Dragsted L, Barri T, Overvad K, Bach Knudsen KE, Hedemann MS, Arner P, Dahlman I, Borge GIA, Baardseth P, Ulven SM, Gunnarsdottir I, Jónsdóttir S, Thorsdottir I, Orešič M, Poutanen KS, Risérus U, Akesson B (2013). Effects of an isocaloric healthy Nordic diet on insulin sensitivity, lipid profile and inflammation markers in metabolic syndrome—a randomized study (SYSDIET). J Intern Med.

[12] Kanerva N, Rissanen H, Knekt P, Havulinna AS, Eriksson JG, Männistö S (2014). The healthy Nordic diet and incidence of Type 2 Diabetes–10-year follow-up. Diabetes Res Clin Pract.

[13] Lacoppidan SA, Kyrø C, Loft S, Helnæs A, Christensen J, Hansen CP, Dahm CC, Overvad K, Tjønneland A, Olsen A (2015). Adherence to a healthy nordic food index is associated with a lower risk of type-2 diabetes—the Danish Diet, Cancer and Health Cohort Study. Nutrients.

[14] Salonen JT (1988). Is there a continuing need for longitudinal epidemiologic research? The Kuopio Ischaemic Heart Disease Risk Factor Study. Ann Clin Res.

[15] Salonen JT, Salonen R, Seppänen K, Rauramaa R, Tuomilehto J (1991). HDL, HDL2, and HDL3 subfractions, and the risk of acute myocardial infarction. a prospective population study in eastern Finnish men. Circulation.

[16] Salonen JT, Nyyssönen K, Korpela H, Tuomilehto J, Seppänen R, Salonen R (1992). High stored iron levels are associated with excess risk of myocardial infarction in eastern Finnish men. Circulation.

[17] Lakka TA, Venäläinen JM, Rauramaa R, Salonen R, Tuomilehto J, Salonen JT (1994). Relation of leisure-time physical activity and cardiorespiratory fitness to the risk of acute myocardial infarction. N Engl J Med.

[18] Bellou V, Belbasis L, Tzoulaki I, Evangelou E (2018). Risk factors for type 2 diabetes mellitus: an exposure-wide umbrella review of meta-analyses. PLoS One.

[19] Neuenschwander M, Ballon A, Weber KS, Norat T, Aune D, Schwingshackl L, Schlesinger S (2019). Role of diet in type 2 diabetes incidence: umbrella review of meta-analyses of prospective observational studies. BMJ.

[20] InterAct Consortium Anneleen (2015). Dietary fibre and incidence of type 2 diabetes in eight European countries: the EPIC-InterAct Study and a meta-analysis of prospective studies. Diabetologia.

[21] Gijsbers L, Ding EL, Malik VS, de Goede J, Geleijnse JM, Soedamah-Muthu SS (2016). Consumption of dairy foods and diabetes incidence: a dose-response meta-analysis of observational studies. Am J Clin Nutr.

[22] Wang P, Fang J, Gao Z, Zhang C, Xie S (2016). Higher intake of fruits, vegetables or their fiber reduces the risk of type 2 diabetes: A meta-analysis. J Diabetes Invest.

[23] Aune D, Ursin G, Veierød MB (2009). Meat consumption and the risk of type 2 diabetes: a systematic review and meta-analysis of cohort studies. Diabetologia.

[24] Imamura F, O'Connor L, Ye Z, Mursu J, Hayashino Y, Bhupathiraju SN, Forouhi NG (2015). Consumption of sugar sweetened beverages, artificially sweetened beverages, and fruit juice and incidence of type 2 diabetes: systematic review, meta-analysis, and estimation of population attributable fraction. BMJ.

[25] Schwab U, Lauritzen L, Tholstrup T, Haldorsson TI, Riserus U, Uusitupa M, Becker W (2014). Effect of the amount and type of dietary fat on cardiometabolic risk factors and risk of developing type 2 diabetes, cardiovascular diseases, and cancer: a systematic review. Food Nutr Res.

[26] Wu JHY, Marklund M, Imamura F, Tintle N, Ardisson Korat AV, de Goede J, Zhou X, Yang W, de Oliveira O, Marcia C, Kröger J, Qureshi W, Virtanen JK, Bassett JK, Frazier-Wood AC, Lankinen M, Murphy RA, Rajaobelina K, Del Gobbo LC, Forouhi NG, Luben R, Khaw K, Wareham N, Kalsbeek A, Veenstra J, Luo J, Hu FB, Lin H, Siscovick DS, Boeing H, Chen T, Steffen B, Steffen LM, Hodge A, Eriksdottir G, Smith AV, Gudnason V, Harris TB, Brouwer IA, Berr C, Helmer C, Samieri C, Laakso M, Tsai MY, Giles GG, Nurmi T, Wagenknecht L, Schulze MB, Lemaitre RN, Chien K, Soedamah-Muthu SS, Geleijnse JM, Sun Q, Harris WS, Lind L, Ärnlöv J, Riserus U, Micha R, Mozaffarian D (2017). Omega-6 fatty acid biomarkers and incident type 2 diabetes: pooled analysis of individual-level data for 39 740 adults from 20 prospective cohort studies. Lancet Diabetes Endocrinol.

[27] Wu JH, Micha R, Imamura F, Pan A, Biggs ML, Ajaz O, Djousse L, Hu FB, Mozaffarian D (2012). Omega-3 fatty acids and incident type 2 diabetes: a systematic review and meta-analysis. Br J Nutr.

[28] Virtanen JK, Mursu J, Voutilainen S, Uusitupa M, Tuomainen T (2014). Serum omega-3 polyunsaturated fatty acids and risk of incident type 2 diabetes in men: the Kuopio Ischemic Heart Disease Risk Factor study. Diabetes Care.

[29] Yary T, Voutilainen S, Tuomainen T, Ruusunen A, Nurmi T, Virtanen JK (2016). Serum n-6 polyunsaturated fatty acids, Δ5- and Δ6-desaturase activities, and risk of incident type 2 diabetes in men: the Kuopio Ischaemic Heart Disease Risk Factor Study. Am J Clin Nutr.

[30] Facchini F, Chen YD, Reaven GM (1994). Light-to-moderate alcohol intake is associated with enhanced insulin sensitivity. Diabetes Care.

[31] Zimorovat A, Mohammadi M, Ramezani-Jolfaie N, Salehi-Abargouei A (2020). The healthy Nordic diet for blood glucose control: a systematic review and meta-analysis of randomized controlled clinical trials. Acta Diabetol.

[32] Trimigno A, Khakimov B, Savorani F, Poulsen SK, Astrup A, Dragsted LO, Engelsen SB (2020). Human urine 1H NMR metabolomics reveals alterations of the protein and carbohydrate metabolism when comparing habitual average Danish diet vs. healthy new Nordic diet. Nutrition.

[33] de Groot RHM, Emmett R, Meyer BJ (2019). Non-dietary factors associated with n-3 long-chain PUFA levels in humans—a systematic literature review. Br J Nutr.

[34] Olsen A, Egeberg R, Halkjær J, Christensen J, Overvad K, Tjønneland A (2011). Healthy aspects of the Nordic diet are related to lower total mortality. J Nutr.

