# Supplementary material for: Adherence to a healthy Nordic diet and risk of type 2 diabetes among men: the Kuopio Ischaemic Heart Disease Risk Factor Study
Source: Eur J Nutr. 2021 Apr 27;60(7):3927–34. doi: 10.1007/s00394-021-02569-1 (PMC8437904; doi:10.1007/s00394-021-02569-1)
Supplement: Supplementary file 1 — Supplementary file1 (DOCX 29 KB) [file 394_2021_2569_MOESM1_ESM.docx]

**European Journal of Nutrition**

**Adherence to a healthy Nordic diet and risk of type 2 diabetes among men: the Kuopio Ischaemic Heart Disease Risk Factor Study**

Hanna-Mari Tertsunen^1^, Sari Hantunen^1^, Tomi-Pekka Tuomainen^1^, Jyrki K. Virtanen^1^

^1^University of Eastern Finland, Institute of Public Health and Clinical Nutrition, P.O. Box 1627, 70211 Kuopio, Finland.

**Corresponding author**: Jyrki K Virtanen, University of Eastern Finland, Institute of Public Health and Clinical Nutrition, Kuopio, Finland. Phone: +358-40-3552957, E-mail: jyrki.virtanen@uef.fi.

| **Supplemental table 1** The components of the original Baltic Sea Diet Score and those used in the present study and the cut-off values for component intakes | | | |
| --- | --- | --- | --- |
|  | |  | Healthy Nordic diet score^a^ |
| Score Component | Contents of the original Baltic Sea Diet Score [1] | Contents in the current study | Cut-off values in the current study |
| Fruits and berries (g/d) | Berries, apples, pears | All fruits, berries | 39; 105; 188 |
| Vegetables (g/d) | Tomato, cucumber, cabbage, roots, peas, lettuce | Roots, pulses, vegetables | 62; 103; 159 |
| Cereals (g/d) | Rye, oats, barley | Whole grains^b^ | 109; 149; 199 |
| Low-fat milk (g/d) | Fat-free milk and milk <2% fat | Fat-free milk and milk <2% fat | 77; 218; 474 |
| Fish (g/d) | Salmon, freshwater fish | Salmon, freshwater fish | 0; 29; 63 |
| Meat products (g/d) | Beef, pork, processed meat products, sausages | Processed and unprocessed meat | 87; 125; 179 |
| Total fat (E%^c^) | Total fat as a percentage of total energy intake | Total fat as a percentage of total energy intake | 35; 39; 42 |
| Fat ratio | Ratio of PUFA^d^ to SFA^e^ + trans-fatty acids | Ratio of PUFA to SFA + trans-fatty acids | 0.16; 0.23; 0.31 |
| Alcohol (g/d)^f^ | Ethanol | Ethanol | 20 |

^a^The healthy Nordic diet score was calculated using the population-based consumption quartiles as cut-offs, with each intake quartile scored as 0, 1, 2 or 3 points. For the potentially healthy score components (fruits and berries, vegetables, cereals, low-fat milk, fish, and fat ratio), the lowest intake category was given 0 points and the highest 3 points. For the potentially less favourable score components (meat products and total fat), the lowest intake category was given 3 points and the highest 0 points.

^b^Excluding rice and pasta.

^c^Percent of energy.

^d^PUFA, polyunsaturated fatty acids.

^e^SFA, saturated fatty acids.

^f^Men consuming 20 g or less of alcohol per day received 1 point; otherwise 0 points were given.

**References**

1. Kanerva N, Kaartinen NE, Schwab U, Lahti-Koski M, Männistö S (2014) The Baltic Sea Diet Score: a tool for assessing healthy eating in Nordic countries. Public Health Nutr 17:1697-1705
